# Physics-Guided Neural Reconstruction of Cellular Membranes for 3D Electron Microscopy

**DOI:** 10.64898/2026.08.01.742159

**Published:** 2026-08-04

**Authors:** Atsushi Matsuda, Se Min Kim, Matthew Akamatsu, Christopher T. Lee

## Abstract

With advances in three-dimensional electron microscopy modalities, quantitative characterization of membrane ultrastructure has emerged as an approach to interrogate how organization of proteins and other components around the membrane drive structure and function. Hindering these efforts, the confident reconstruction of geometric features such as membrane curvature is challenging since it requires the calculation of higher-order derivatives from discrete membrane representations. Modern advances in using neural networks to learn continuous implicit representations of complex shapes present a promising solution to this problem. This work presents a physics-informed neural network framework for reconstructing membrane geometries to curvature-order accuracy from images using an implicit neural representation. Benchmarking using synthetic data illustrates that physics-based regularization during training improves accuracy of recovered curvatures, improving robustness to image noise. Application to experimental datasets demonstrate that the framework generalizes to complex cellular structure, such as the Golgi apparatus and mitochondria. We further perform three-dimensional curvature analysis of endocytic pits in cells to reveal anisotropic curvatures at the pit neck, previously predicted to be a lower-energy pathway for neck constriction. This work provides a unified framework for reconstructing three-dimensional membrane shape, including curvature, from volumetric imaging data. By capturing membrane geometry more accurately, our approach yields mechanical insights that can be linked to molecular-scale interactions.

## Full Text Availability

The license terms selected by the author(s) for this preprint version do not permit archiving in PMC. The full text is available from the preprint server.

